# Efficacy study of olmesartan medoxomil on coronary atherosclerosis progression and epicardial adipose tissue volume reduction in patients with coronary atherosclerosis detected by coronary computed tomography angiography: study protocol for a randomized controlled trial

**DOI:** 10.1186/s13063-015-1097-z

**Published:** 2016-01-06

**Authors:** Ying Zhou, Feng Tian, Jing Wang, Jun-Jie Yang, Tao Zhang, Jing Jing, Yun-Dai Chen

**Affiliations:** Department of Cardiology, Chinese PLA General Hospital, Beijing, 100853 China

**Keywords:** Coronary atherosclerosis, Coronary computed tomography angiography, Epicardial adipose tissue

## Abstract

**Background:**

Epicardial adipose tissue (EAT) is a newly discovered independent risk factor for coronary atherosclerosis. There is a scarcity of information on the reduction of EAT volume to reduce atherosclerosis risk. Coronary computed tomography angiography (CCTA) has emerged as a noninvasive imaging method for the analysis of coronary atherosclerosis and EAT volume. The purpose of this trial is to determine whether olmesartan medoxomil is effective at both treatment of coronary atherosclerosis progression and EAT volume reduction in patients with coronary atherosclerosis detected by CCTA.

**Methods/design:**

This study is a prospective, single-center, open-label, randomized controlled clinical trial aimed at exploring the efficacy of olmesartan medoxomil on coronary atherosclerosis and EAT. A total of 194 patients with coronary stenosis greater than 30 % and less than 70 % detected by CCTA will be randomly divided into olmesartan medoxomil or conventional antihypertensive medication groups (1:1 ratio). The primary outcome measures include coronary atherosclerosis progression and EAT volume reduction, as detected by CCTA at 12 months. The secondary outcome measures include the levels of blood lipids, glucose, high-sensitivity C-reactive protein, IL-6, monocyte chemotactic protein 1, TNF-α, matrix metalloproteinase 9, NO, endothelin 1, adiponectin, and leptin at baseline and after 6 and 12 months.

**Discussion:**

Treatments aimed at reducing EAT volume can eventually achieve an antiatherosclerotic effect. This is the first trial designed to explore the effect of olmesartan medoxomil on both coronary atherosclerosis progression and EAT volume reduction in patients with coronary atherosclerosis detected by CCTA.

**Trial registration:**

ClinicalTrials.gov: NCT02360956.

## Background

### Epicardial adipose tissue and coronary atherosclerosis

Epicardial adipose tissue (EAT) is directly deposited around the pericardium and coronary artery. By autocrine and paracrine means, EAT can generate various kinds of cytokines, inflammatory mediators and free fatty acids. These biological indicators can affect the state of coronary endothelial function and promote inflammation and oxidative stress, which finally aggravate the progression of coronary atherosclerosis [1–3]. A significant amount of clinical research has been conducted to investigate the link between EAT and coronary atherosclerosis. It was claimed that calcified plaque progression in patients without coronary artery disease and in patients with type 2 diabetes mellitus were associated with a larger EAT volume [4, 5]. Epicardial adipose tissue is also an independent predictor of significant coronary stenosis and is independently associated with high-risk coronary plaque features, such as low CT attenuation plaque, thin-cap fibroatheroma and positive remodeling [6]. Not only is EAT, as a special visceral fat, correlated with the increased development of coronary artery atherosclerosis, but it is also associated with adverse coronary events [7].

### Treatment of epicardial adipose tissue and coronary atherosclerosis

There are ample studies exploring the progression of coronary atherosclerosis following pharmacological manipulation. At the time of writing, the use of statins is recognized as an effective treatment; statins can result in decreases in plaque and necrotic core volume, and can also significantly reduce the progression of low attenuation plaque (<30 Hounsfield units) and non-calcified plaque [8, 9]. Other drugs, such as dipeptidyl-peptidase 4 inhibitors [10], a PPARγ agonist (pioglitazone) [11], atorvastatin plus ezetimibe [12], olmesartan [13], have also been reported to have antiatherosclerotic effects, although the effects have not been verified by large-scale studies. As studies show that EAT volume is associated with plaque progression and cardiovascular adverse events, treatments aimed at reducing EAT volume may finally achieve an antiatherosclerotic, preventive effect. However, at the time of writing, limited studies have aimed at reducing both EAT and plaque volume to achieve an antiatherosclerotic effect. A serial coronary computed tomography angiography (CCTA) study recently indicated that intensive statin therapy can reduce the EAT volume of Europeans, but the study failed to demonstrate a relationship among EAT volume reduction, coronary atherosclerosis progression and clinical prognosis [14]; moreover, intensive statin therapy might not be appropriate for Asians. The epidemiological studies and clinical researches show that Asians may have poorer tolerability and safety to intensive statins than white people, owing to genetic differences (variants in structure or polymorphisms) in pharmacokinetics and pharmacodynamics properties [15–17]. It has been claimed that polymorphic variants in cytochrome P450 (CYP450) families that were associated with statin metabolism might result in varying rates of metabolic clearance. CYP450 2C19 slow metabolizer phenotype was reported to be present in approximately 16 % of Asians compared with only about 3 % of white people [18]. Polymorphic variants in the predominant CYP450 isoform, CYP450 3A4, were reported to be associated with a functional decrease in the enzyme’s activity in dyslipidemic Chinese patients [19]. The HPS2-THRIVE study recently also indicated that, using same-dose statin treatment, an excess of increased alanine aminotransferase was seen mainly among Chinese patients (more than three consecutive values above the upper limit of normal of 0.24 %/year compared with 0.02 %/year in Europe) [20]. Moreover, the morbidity of chronic hepatitis B was high in china with nearly 90 million infections. For these reasons, intensive statin therapy may result in higher hepatotoxicity in Asian populations than in white populations, so low- to moderate- dose statin therapy might be more appropriate for Asian populations [15, 16]. Subjects who undergo weight loss exercise, bariatric surgery, or low-dose aspirin therapy can also reduce EAT volume or inflammation, but the effects are weak and these treatments cannot achieve good results in patients with coronary atherosclerosis progression [21–24]. Our aim is to find a drug that reduces EAT volume while inhibiting the progression of coronary atherosclerosis.

In recent years, studies have confirmed that olmesartan medoxomil can improve endothelial function, resist thrombosis, improve tissue reconstruction, and resist oxidative stress to achieve atherosclerosis resistance [13, 25–28]. The latest research shows that olmesartan medoxomil can better inhibit rat epididymal adipose cell hypertrophy and inflammatory reactions [29]. Therefore, we hypothesized that olmesartan medoxomil may also reduce EAT volume, finally achieving an anti-atherosclerosis effect.

### EAT and coronary atherosclerosis imaging with computed tomography

Compared with such invasive methods as intravascular ultrasound, virtual histology intravascular ultrasound, optical coherence tomography, and fractional flow reserve, CCTA has emerged as a noninvasive imaging method that analyzes both coronary atherosclerosis and EAT volume [14, 30]. To date, ample CCTA studies have explored the progression of coronary atherosclerosis following pharmacological manipulation. Thus, in this study, using CCTA as a noninvasive method to analyze both coronary atherosclerosis progression and EAT volume is of significant clinical value.

#### Aims of the main study

The purpose of this study is to determine whether olmesartan medoxomil is effective on both the treatment of coronary atherosclerosis progression and EAT volume reduction in patients with coronary atherosclerosis detected by CCTA.

#### Aims of the anti-atherosclerosis mechanism study

1. To explore the relationship between coronary atherosclerosis progression and EAT volume reduction.
2. To explore the effect of olmesartan medoxomil on serum levels of blood lipids, glucose, circulating surrogate markers of atherosclerosis inflammation, including high-sensitivity C-reactive protein, IL-6, monocyte chemotactic protein 1 (MCP-1), TNF-α, and matrix metalloproteinase 9 (MMP-9), circulating surrogate markers of endothelial function, including NO and endothelin 1 (ET-1), and circulating surrogate markers of adipose tissue inflammation and metabolism, including adiponectin and leptin at baseline and after 6 and 12 months.

## Methods/design

### Study design

**Fig. 1:**
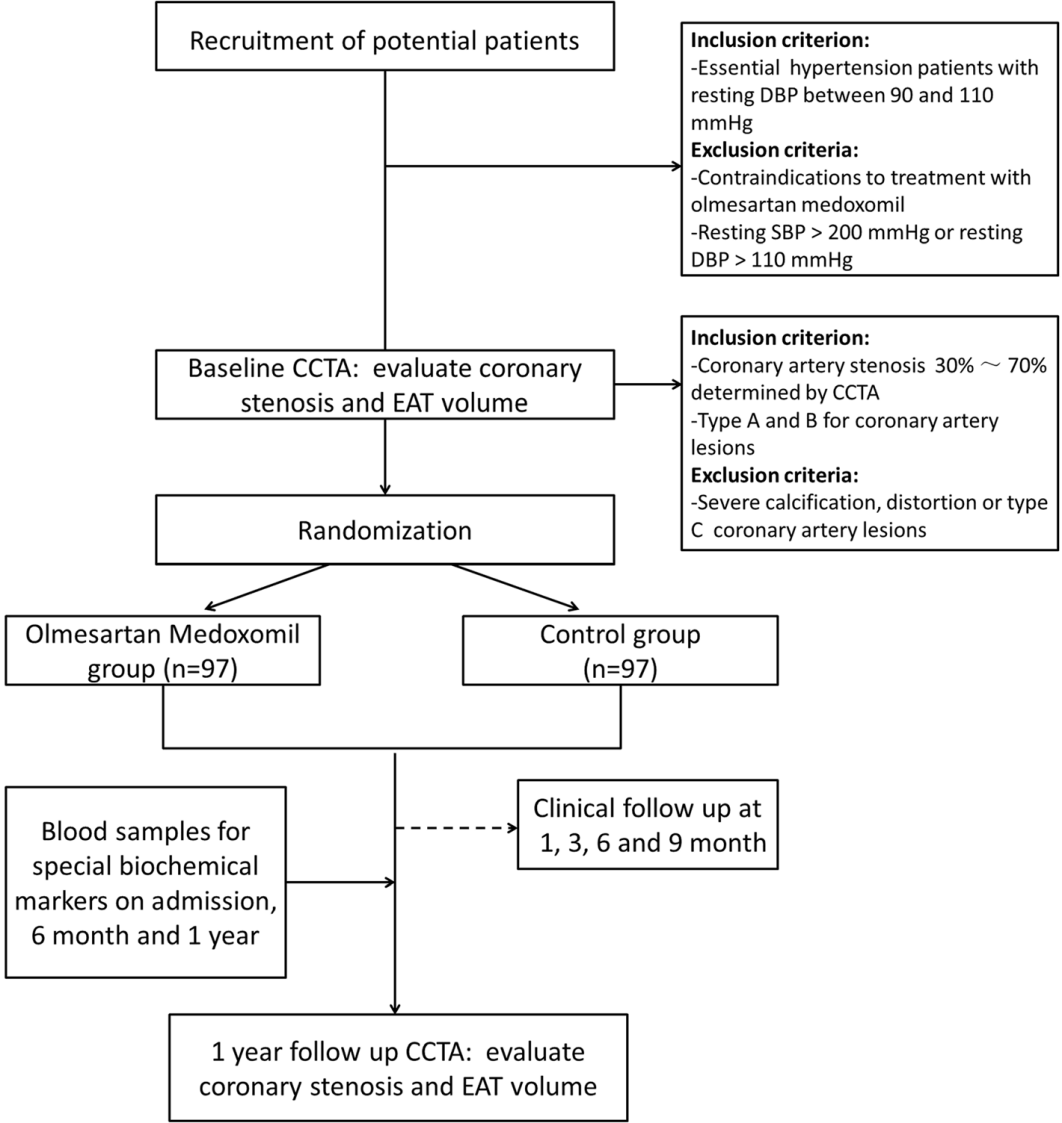
Study flowchart. CCTA, coronary computed tomography angiography; DBP, diastolic blood pressure; EAT, epicardial adipose tissue; SBP, systolic blood pressure

This study is a prospective, single-center (Chinese PLA General Hospital, Beijing, China), open-label, randomized controlled trial of the efficacy of olmesartan medoxomil on coronary atherosclerosis and EAT. Consecutive patients with coronary stenosis greater than 30 % and less than 70 % detected by CCTA will be randomly assigned to olmesartan medoxomil or conventional antihypertensive medication groups (1:1 ratio). Coronary computed tomography angiography will be conducted at the Department of Cardiology (Chinese PLA General Hospital). Primary outcome measures include coronary atherosclerosis progression and EAT volume reduction, as detected by CCTA, at 12 months. Secondary outcome measures include levels of blood lipids, glucose, high-sensitivity C-reactive protein, IL-6, MCP-1, TNF-α, MMP-9, NO, ET-1, adiponectin, and leptin at baseline and after 6 and 12 months. The study design is summarized in Fig. 1.

### Clinical inclusion and exclusion criteria

Clinical inclusion criteria are:

- Age between 18 and 75 years;
- Coronary artery stenosis between 30 % and 70 % determined by CCTA in essential hypertension patients;
- Resting diastolic blood pressure between 90 and 110 mmHg;
- Type A and B for coronary artery vascular lesions.

Clinical exclusion criteria are:

- Secondary hypertension;
- Coronary artery stenosis less than 30 % or greater than 70 %, as determined by CCTA;
- Severe arrhythmia;
- Severe cardiac insufficiency or left ventricular dysfunction (left ventricular ejection fraction < 30 %);
- Severe hepatic or kidney insufficiency;
- Resting systolic blood pressure > 200 mmHg or resting diastolic blood pressure >110 mmHg;
- Contraindications to treatment with olmesartan medoxomil (allergy, glaucoma, digestive ulcer, currently taking phosphodiesterase-5 inhibitor);
- Severe calcification, distortion or type C coronary artery vascular lesions;
- Pregnancy;
- Unwillingness or inability to provide informed consent.

### Randomization

Information regarding the study will be provided to the patient at the Department of Cardiology. Once informed consent is obtained, the patient will be randomized at the Department of Cardiology. Subjects will be randomized to either olmesartan medoxomil or conventional antihypertensive medication groups (1:1 ratio). Participants will be randomized before the first treatment using a blocked randomization procedure (computerized random numbers) and will incorporate minimization to ensure matching for age, sex, body mass index, and hypertension grade.

### Ethical considerations

The Chinese PLA General Hospital Ethics Committee approved this study on 12 December 2014 (reference number S2014-119-01). This study complies with the Declaration of Helsinki. Informed consent will be obtained from all participating patients. Upon signing informed consent, patients’ data will be populated as per protocol.

### Details of CCTA examination

#### CCTA examination procedure

CCTA will be performed on a dual-source CT scanner (Somatom Definition Flash, Siemens Healthcare, Forchheim, Germany). All enrolled patients will be instructed in the breath-holding technique before CCTA to minimize breathing artifacts. Three minutes before CCTA, all patients will be given 0.5 mg nitroglycerin sublingually to dilate the coronary artery. The scan range is from the carina or the pulmonary artery segment down to 1 cm below the diaphragm. Electrocardiography is continuously performed throughout the entire examination for each patient. A dual-head power injector (SCT 210, Medrad, USA) and a nonionic contrast medium (Ultravist®, 370 mg I /ml, Schering AG, Guangzhou, China) will be used. The collimation is 2 × 128 × 0.6 mm, the gantry rotation time is 0.28 ms, the slice thickness is 0.6 mm, the tube voltage is 80 to 120 kV (modified using a care kV, Siemens Medical Solutions, Forchheim, Germany), and the tube current is 290 to 560 mAs/rotation (scout-based automatic reference tube current selection – CareDose 4D, Siemens Medical Solutions, Forchheim, Germany). For double flash acquisition, the pitch is 3.4, and for retrospectively ECG-triggered spiral acquisition, the pitch will vary depending on the patient’s heart rate. Total estimated radiation dose for the patient will be recorded.

Patients with a heart rate ≤70 beats/min will be evaluated using double prospective ECG-gated high-pitch CT angiography (double flash mode). For the double flash protocol, the contrast-enhanced CCTA protocol is as follows: a test bolus scan will be performed at the level of the aortic root with administration of 15 ml of contrast medium into the right antecubital vein at a rate of 5.0 ml/s, followed by an injection of 20 ml of saline flush at the same flow rate to obtain a peak enhancement time curve. The double flash acquisition triggered scan time is 4 s after the peak enhancement time. After calculating the triggered scan time, double flash acquisition will be performed by injecting 60 to 90 ml contrast medium at a rate of 5.0 ml/s, immediately followed by 35 ml of 70/30 contrast/saline material mixture and 50 ml saline bolus at the same flow rate. The first scan will begin at 60 % of the R-R interval from the craniocaudal direction. The second scan will be acquired at 30 % of the R-R interval 3 s after the first scan during the same contrast injection time.

Patients with a heart rate >70 beats/min will be evaluated using retrospectively ECG-triggered spiral acquisition. The retrospective protocol is as follows: 60 to 90 ml contrast medium is injected into the antecubital vein at a rate of 5.0 ml/s, immediately followed by 50 ml saline solution at the same flow rate. Bolus tracking is used, and the region of interest is set at the root of the ascending aorta. We will perform the scan with a delay of 5 s after the root of the ascending aorta reaches a threshold of 100 Hounsfield units. For this scan mode, the acquisition is from 30 % to 80 % of the R-R interval.

#### CCTA image post-processing

All CCTA data will be sent to the Syngo Multi-Modality Workplace for post-processing. Two independent experienced observers who are unaware of the patients’ clinical information will evaluate the CCTA data in different modes, including maximum intensity projection, volume rendering, curved-planar reconstructions, and the original transaxial images. Disagreements in data analysis between the two readers will be resolved by consensus reading.

#### Epicardial adipose tissue quantification

The EAT volume will be measured by two experienced radiologists using the same sets of images acquired for the CCTA. The radiologists will be blinded to the purpose of the study, clinical characteristics and patients’ anthropometric data.

The EAT volume is defined as the total amount of adipose tissue deposited between the surface of the heart and the visceral pericardium. The region of interest in measuring the EAT volume includes the heart and the surrounding EAT. By manually tracing the epicardium contours in the axial slices from the bifurcation of the pulmonary artery to the diaphragm, the EAT volume is analyzed. The pericardium contour is traced every 10 mm, from the lower visible level of the pulmonary artery bifurcation until the top level of the pulmonary valve, for every 20 mm until the first slice where the diaphragm becomes visible, and for every 10 mm from this point until the last slice where the pericardium is still visible [31]. The pericardium contour is manually outlined by the radiologists, and then the software (Syngo Volume, Siemens Medical Solutions) automatically calculates the total EAT volume. Computed tomography attenuation ranging from −195 to 45 Hounsfield units is applied to isolate the EAT from other tissues. Mediastinal adipose tissue and pericardial adipose fat (fat deposit outside the visceral pericardium and on the external surface of the parietal pericardium) are excluded from the analysis. For the assessment of interobserver agreement, we will randomly select 50 patients, and all EAT measures will be assessed by two experienced radiologists blinded to the other radiologist’s measurements.

#### Definition of coronary atherosclerosis plaque progression

We will use QAngio CT post-processing software (QAngioCT Research Edition version 2.1.0, Medis Medical Imaging Systems, Leiden, the Netherlands) to evaluate all CCTA data. All three vessels will be assessed in each patient using the 15-segment American Heart Association model for coronary segment classification [32]. Only segments with a diameter ≥2.0 mm and without stent implantation will be considered for analysis. Parameters including minimal lumen diameter, percent diameter stenosis, minimum lumen area, plaque burden, plaque volume, vascular remodeling index, plaque type classification (calcification, necrosis, fiber, and fiber lipid plaques) based on segments will be analyzed using QAngio CT post-processing software.

Coronary atherosclerosis progression is defined as ≥10 % diameter reduction or progression of a pre-existing coronary stenosis or ≥0.2 mm reduction or progression of the minimal luminal diameter in the lesion [30].

### Medication intervention and control protocols

All patients should accept lifestyle interventions and conventional anti-atherosclerosis treatment. The low-density lipoprotein cholesterol level should be controlled below 100 mg/dl, and the blood pressure should be controlled below 140/90 mmHg.

In the olmesartan medoxomil group, the usual recommended starting dose of olmesartan medoxomil is 20 mg once daily when used as monotherapy in patients who are not volume-contracted. For patients requiring further reduction in blood pressure after 2 weeks of therapy, the dose of olmesartan medoxomil may be increased to 40 mg. Doses above 40 mg do not appear to have a greater effect. Twice-daily dosing offers no advantage over the same total dose given once daily.

In the control group, any antihypertensive medication alone or in combination, including calcium channel blockers, diuretics, beta blockers, or other antihypertensive medication except angiotensin-converting enzyme inhibitors or angiotensin II receptor blockers can be used. The drug dose must be individualized. The patients should take the antihypertensive drugs according to the doctors’ recommendations.

### Study outcomes

The primary outcome measures are coronary atherosclerosis progression and EAT volume changes, as detected by CCTA at 12 months.

The secondary outcome measures include

1. The relationship between coronary atherosclerosis and EAT;
2. Serum levels of blood lipids, glucose, circulating surrogate markers of atherosclerotic inflammation including high-sensitivity C-reactive protein, IL-6, MCP-1, TNF-α, and MMP-9, individual circulating surrogate markers of endothelial function including NO and ET-1, and individual circulating surrogate markers of adipose tissue inflammation and metabolism including adiponectin and leptin at baseline and after 6 and 12 months.

### Biomarkers

Two 10-ml samples of blood will be collected from the antecubital vein by a trained nurse for each individual. Fasting blood samples will be obtained between 7:00 a.m. and 12:00 noon to control for possible diurnal variations. Blood samples will be centrifuged at 4 °C and 3,000 rpm for 15 min. The serum will be sampled and stored at −80 °C until analysis. Traditional cardiovascular blood risk markers, including fasting blood glucose, triglycerides, total cholesterol, and high- and low-density lipoprotein cholesterol will be assessed. The following proinflammatory markers will be assessed: high-sensitivity C-reactive protein, IL-6, TNF-α, MCP-1, and MMP-9. In addition, NO and ET-1 will be measured, to assess endothelial function. Markers of adipose tissue inflammation and metabolism, including adiponectin and leptin, will also be assessed. These markers will be measured at baseline and 6 and 12 months after treatment.

### Follow-up

Clinical follow-up will take place at 1 month (±1 week), 3 months (±2 weeks), 6 months (±2 weeks), 9 months (±30 days), and 1 year (±30 days) by clinical visit or phone interview.

At baseline and 6-month (±2 weeks) and 1 year (±30 days) follow-ups, all patients will provide venous blood for detection of blood lipids, glucose, high-sensitivity C-reactive protein, IL-6, MCP-1, TNF-α, NO, ET-1, MMP-9, adiponectin, and leptin.

At 1 year (±30 days) follow-up, all patients will undergo CCTA (with QAngio CT post-processing software).We anticipated a patient drop-out rate of 10 %.

### Sample size calculation

This trial is an open-label randomized clinical trial, so patients will randomly be assigned to olmesartan medoxomil or conventional antihypertensive medication groups (1:1 ratio). The purpose of this study is to verify that olmesartan medoxomil is effective in the treatment of coronary atherosclerosis progression and EAT volume reduction in patients with coronary atherosclerosis detected by CCTA. We also want to elucidate the relationship between coronary atherosclerosis and EAT. The mechanism by which olmesartan medoxomil inhibits coronary atherosclerosis progression will be studied by detecting the serum levels of blood lipids, glucose, circulating surrogate markers of atherosclerosis inflammation including high-sensitivity C-reactive protein, IL-6, MCP-1, TNF-α, and MMP-9, circulating surrogate markers of endothelial function, including NO and ET-1, and circulating surrogate markers of adipose tissue inflammation and metabolism, including adiponectin and leptin.

Studies on coronary atherosclerosis progression rate have had differing results. The combined results of multiple studies indicate that the conventional mean coronary atherosclerosis progression rate is about 30 % [33–35]. We hypothesize that additional olmesartan medoxomil use will reduce the coronary atherosclerosis progression rate to 13 % [36–40]. Using double-side inspection, α = 0.05, β = 0.2, we calculate a total sample size of 176 cases; considering the expected loss to follow-up to be 10 %, the number of cases to be included should be at least 176 × (1 + 10 %) = 194. Therefore, we aim for 97 cases of each group.

### Statistical analysis

Continuous variables will be described using means and standard deviations or median and range in case of asymmetric distribution of data. Categorical variables will be presented using frequency distribution. Univariate analyses will be conducted using chi-square and *t* tests for independent samples. A multiple logistic regression analysis will be performed to correlate coronary atherosclerosis progression with clinical variables and EAT volume, including treatment groups. Statistical significance will be considered for *P* < 0.05. A statistical package (SPSS 16.0) will be used for analysis. The individual will be considered the unit of analysis.

## Discussion

To date, there are ample CCTA studies exploring the progression of coronary atherosclerosis following pharmacological manipulation. As studies show that EAT volume is associated with plaque progression and cardiovascular adverse events, treatments aimed at reducing EAT volume may finally achieve an antiatherosclerotic, preventive effect. However, at the time of writing, only a limited number of studies have aimed to reduce both EAT and plaque volume to achieve a preventive effect against atherosclerosis. The novelty of this study is that we intend to explore the effect of olmesartan medoxomil on both plaque volume and epicardial fat. This study will accomplish two goals: (1) it will explain the relationship between EAT volume and coronary atherosclerosis progression and (2) it will verify the effect of olmesartan medoxomil on EAT volume reduction and coronary atherosclerosis progression.

If these hypotheses are supported, the study findings will have significant implications related to clinical practice. Evidence that olmesartan medoxomil is effective on EAT volume reduction and coronary atherosclerosis progression would be very attractive to clinicians and patients. This may further contribute to the care of patients with coronary heart disease.

## Trial status

Recruitment for the study is currently ongoing. Patient recruitment began in December 2014.

## Abbreviations

CCTA: coronary computed tomography angiography
CT: computed tomography
CYP450: cytochrome P450
EAT: epicardial adipose tissue
ET-1: endothelin 1
IL-6: interleukin 6
MCP-1: monocyte chemotactic protein 1
MMP-9: matrix metalloproteinase 9
TNF-α: tumor necrosis factor α
